# Dynamics of Social Behavior in Fruit Fly Larvae

**DOI:** 10.1371/journal.pone.0095495

**Published:** 2014-04-16

**Authors:** Zachary Durisko, Rebecca Kemp, Rameeshay Mubasher, Reuven Dukas

**Affiliations:** 1 Animal Behaviour Group, Department of Psychology, Neuroscience & Behaviour, McMaster University, Hamilton, Ontario, Canada; 2 Social Aetiology of Mental Illness (SAMI) CIHR Training Program, Centre for Addiction and Mental Health, Toronto, Ontario, Canada; Center for Genomic Regulation, Spain

## Abstract

We quantified the extent and dynamics of social interactions among fruit fly larvae over time. Both a wild-type laboratory population and a recently-caught strain of larvae spontaneously formed social foraging groups. Levels of aggregation initially increased during larval development and then declined with the wandering stage before pupation. We show that larvae aggregated more on hard than soft food, and more at sites where we had previously broken the surface of the food. Groups of larvae initiated burrowing sooner than solitary individuals, indicating that one potential benefit of larval aggregations is an improved ability to dig and burrow into the food substrate. We also show that two closely related species, *D. melanogaster* and *D. simulans*, differ in their tendency to aggregate, which may reflect different evolutionary histories. Our protocol for quantifying social behavior in larvae uncovered robust social aggregations in this simple model, which is highly amenable to neurogenetic analyses, and can serve for future research into the mechanisms and evolution of social behavior.

## Introduction

Social behavior can have enormous impacts on the fitness and evolution of animals [1], [2], but its neurogenetic underpinnings and the mechanisms by which it evolves are only beginning to be understood [3], [4]. Crucial for such research is the use of simple animal models [5], [6], and to this end we have investigated social interactions among fruit fly larvae, *Drosophila melanogaster.* Fruit fly larvae are an ideal model system owing to their simple brains, which contain only a few thousand functional neurons [7], [8], and amenability to neurogenetic manipulation. While studies on fruit fly larvae have been immensely successful in furthering our understanding of foraging, locomotion, and the mechanisms of taste, olfaction, and learning [9]–[12], the study of larval social behavior is just beginning. Wu et al. [13] noted that older (wandering stage) larvae are more ‘clumpy’ and seem to engage in cooperative burrowing, adopting a vertical drilling motion, which they suggested may help larvae locate safer sites to pupate, although this remains to be studied closely. Highlighting the utility of the larval model, some of the neural mechanisms involved in this social burrowing were also identified [13], [14]. Recently, it has been shown that larvae are attracted to the visual cues of other ‘writhing’ larvae [15]. In our laboratory, controlled tests with feeding-stage third instar larvae indicated that focal larvae are attracted to groups of other foraging larvae and learn to prefer novel cues previously experienced in the presence of others over novel cues paired with non-social settings [16]. Finally, larvae may use social cues, including different chemical cues between species, to identify adequate sites for pupation [17].

Typically, females lay clusters of eggs on exposed sections of rotting fruit that draw additional females due to: the attractive volatiles of the fruits, the deposition of attractive pheromones, transferred yeast species, and the attractive odor of larval residues [18]–[23]. The resulting aggregations of eggs mean that emerging larvae are likely to have frequent encounters with other larvae, allowing ample opportunity for social interaction. Here we used a novel protocol to quantify the dynamics of social interactions among larvae throughout development from emergence until pupation. Specifically, we asked whether larvae spontaneously form social groups, quantified their level of sociality throughout development, and assessed ecological factors that may affect social behavior. Additionally, we investigated one potential benefit of larval sociality: improved digging ability.

## Results

### Dynamics of Larval Social Behavior

**Figure 1:**
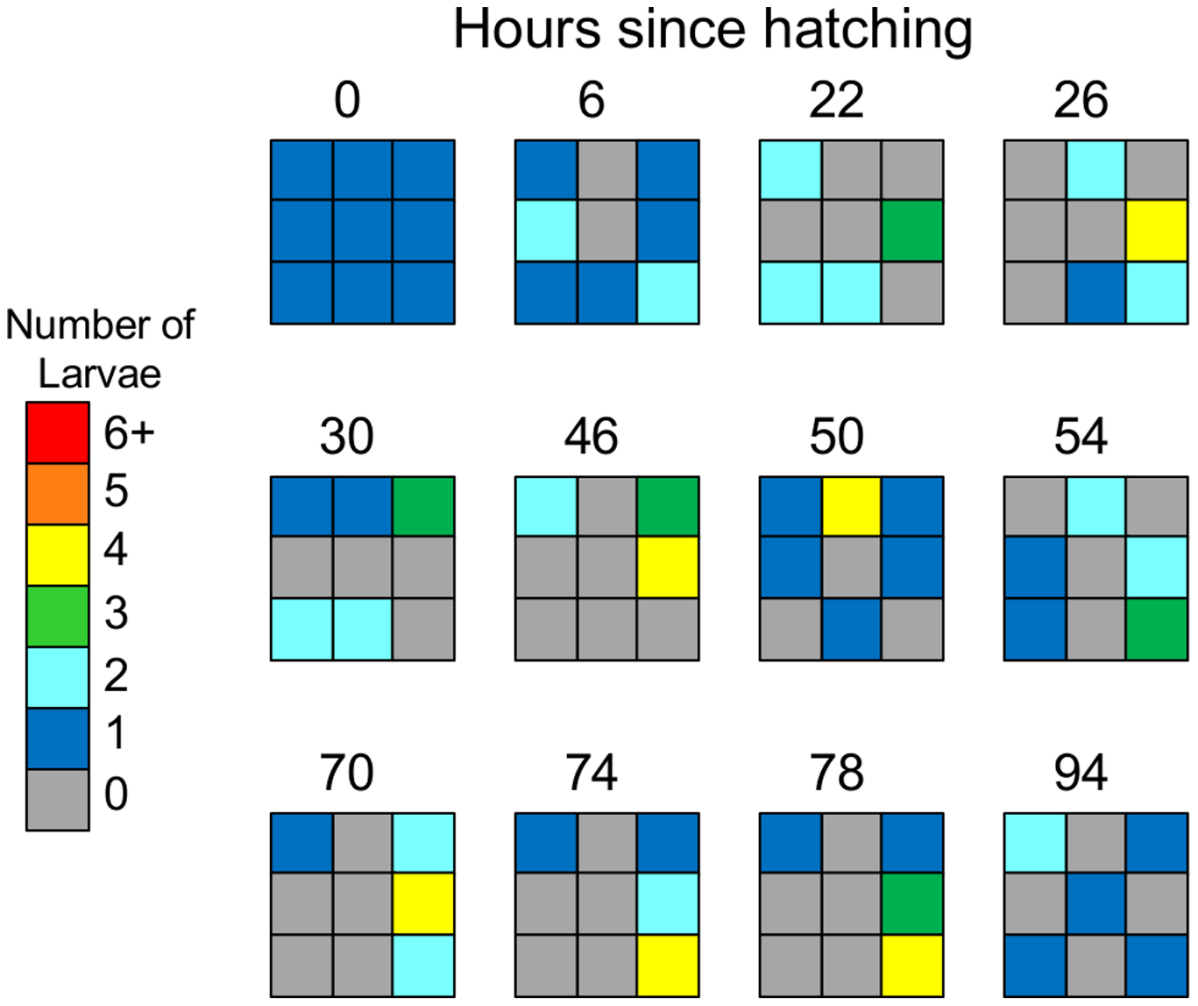
An example dish showing typical larval aggregation and movement behavior starting with a uniform egg distribution. Larvae are highly mobile and form modest aggregations that move over time. This dish was chosen because its aggregation index was closest to the median. Quadrat color represents the number of larvae per quadrat.

**Figure 2:**
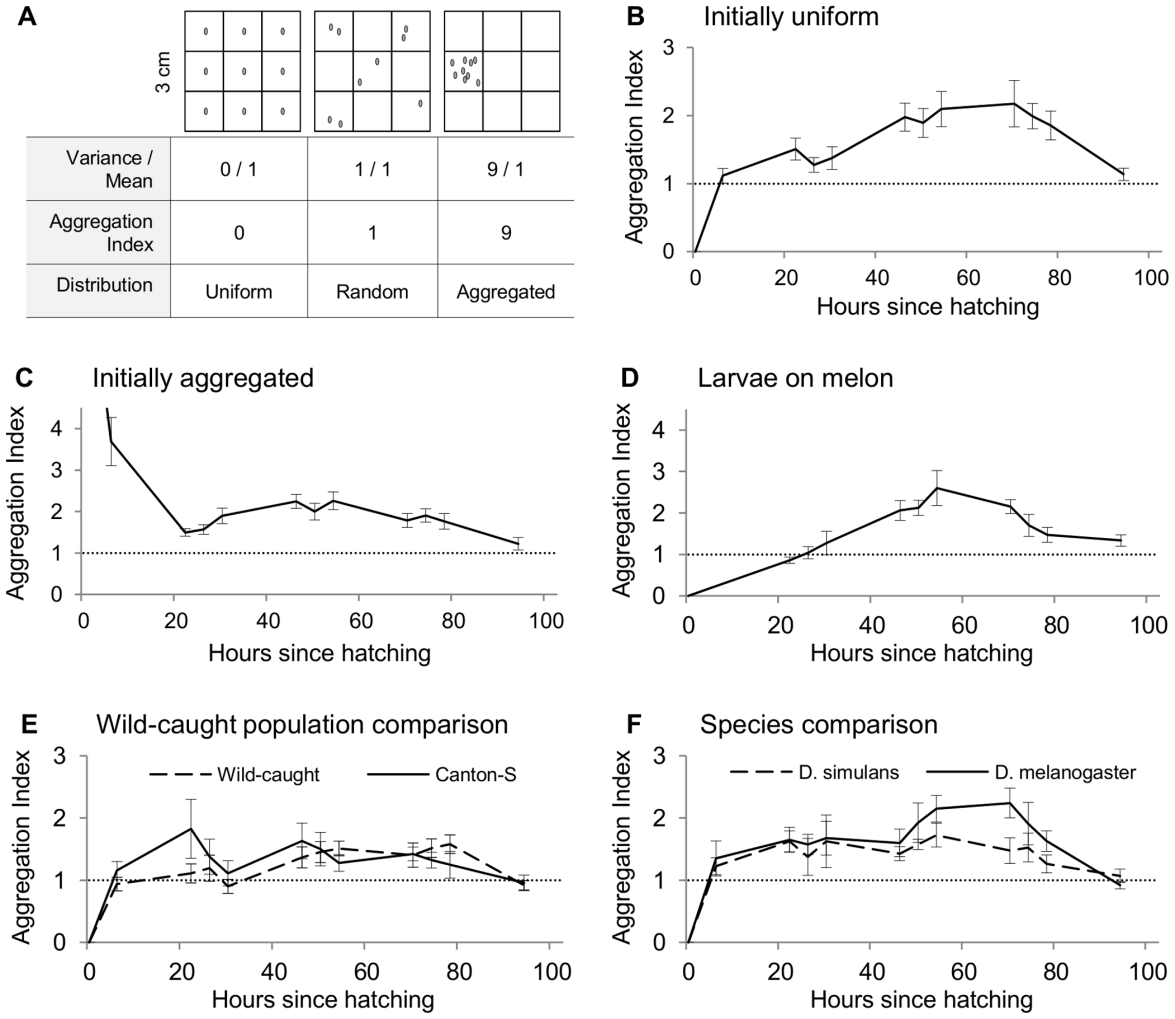
Dynamics of larval aggregation. (**A**) We monitored larval aggregation behavior in 3×3 cm dishes containing nine larvae each, and calculated an aggregation index (variance-to-mean ratio) for each. Indices greater than 1 indicate distributions that are more aggregated than expected by chance. We started the larvae in either a (**B**) perfectly uniform (N = 20, as in the left side of panel A) or (**C**) perfectly aggregated distribution (N = 20, as in the right side of panel A). Lines indicate mean ±1 SE. We also assessed larval aggregation under more naturalistic conditions, with (**D**) larvae reared on a natural melon substrate (N = 10), and (**E**) a comparison between our laboratory strain (N = 9) and an Ontario population of the same species (N = 10). Finally, (**F**) we compared the aggregation behavior of two closely related species, *D. melanogaster* and *D. simulans* (N = 10 for each).

We sought first to document the pattern of social aggregation among larvae from egg until pupation. We monitored the location of nine larvae in square dishes of food by noting the number of larvae within each of nine equally-sized superimposed quadrats (Fig. 1). To quantify aggregation, we calculated an *Aggregation Index*, defined as the variance-to-mean ratio of larvae per quadrat [24]. Random motion is indicated by a value of 1, and indices greater than 1 indicate aggregated or “clumpy” distributions (Fig. 2A). As an additional descriptive measure, we noted the maximum for each dish and report the average maximum aggregation index for each assay. We began with a series of aggregation assays in which we varied (1) the initial distribution of eggs in the assay dish, (2) the food substrate, (3) the population of *D. melanogaster* (comparing our laboratory stock of wild type *Canton S* to a population founded by recently-caught Ontario flies), and (4) the species of fruit fly (comparing *D. melanogaster CS* to *D. simulans*).

In our first assays, we tested larvae that had hatched from one of two initial egg distributions, either uniformly distributed (one egg per quadrat) or perfectly aggregated (all eggs in one quadrat). In both conditions, larvae showed aggregation behavior that peaked between 40–80 h after hatching (Fig. 1; Fig. 2B & C). The aggregation indices of larvae placed in the two different initial distributions rapidly converged within the first 22 h after hatching, after which larvae showed a similar pattern of aggregation behavior throughout the larval stage (Main effect of initial distribution after 22 h, GEE: χ^2^_1_ = 1.6, N = 40, p = 0.209; Interaction between initial distribution and time: χ^2^_9_ = 10.4, p = 0.321; Fig. 2B & C), and both showed a similar significant quadratic trend (Effect of time, GEE: χ^2^_9_ = 81.1, p<0.001; Quadratic: χ^2^_1_ = 53.4, p<0.001; Fig. 2B & C). Aggregation increased throughout the second and third instar stages before declining coincident with the onset of larval wandering prior to pupation. After 22 h, the average maximum aggregation index for each dish reached 3.33±0.17, (N = 40, mean ± SEM), corresponding to ∼5 out of 9 (55.6%) individuals in one quadrat.

We confirmed that larvae were not merely collecting at one preferred location in each dish (e.g., corners), or one particular quadrat (e.g., center), which would result in spuriously high aggregation. Larvae did not prefer one area of the dish over others, forming their greatest aggregations in corner (52.5%), side (45.0%) and center (2.5%) quadrats no differently than expected by chance (4∶4∶1 ratio, respectively; χ^2^_2_ = 3.3, p = 0.196). Also, the location of greatest aggregation of each dish did not differ from random, and larvae formed aggregations in all quadrats (Index: 1.24; χ^2^_8_ = 10.0, less than χ^2^_critical_ = 17.5). Finally, larval aggregations moved throughout the experiment (e.g., Fig. 1), and thus cannot readily be explained by attraction to or remaining in one higher quality site (i.e., *Taxis* or *Orthokinesis* [25]). When starting from a uniform distribution, clumps of four or more larvae formed in 2.4±0.2 different quadrats, with the quadrat of aggregation changing locations an average of 1.7±0.3 times per dish. When starting with a perfectly aggregated distribution (dropping the first two time points that had artificially high aggregation), larvae formed clumps of four or more larvae in 2.5±0.2 different quadrats, with the site of aggregation moving 1.9±0.3 times per dish. In 60% of dishes (12/20), larvae formed their first aggregation of four or more larvae in the quadrat where the eggs had hatched. In 25% of dishes, larvae never formed an aggregation at this site after hatching.

### Dynamics of Larval Aggregation on Fruit and in Wild Flies

To confirm that our observed pattern of larval aggregation is a general phenomenon that would occur in nature (rather than simply an artifact of our food recipe or our laboratory population of *Canton S*), we first tested *Canton S* larvae in dishes filled with natural fruit (slices of honeydew melon, *Cucumis melo*), and second, compared patterns of aggregation on standard food between our lab population of wild-type *Canton S* and a population recently founded from naturally occurring flies caught in Southern Ontario, Canada. In these and all subsequent experiments, eggs were initially arranged uniformly in the experimental dishes.

Larvae formed aggregations on melon similarly to those on laboratory food, with a significant negative quadratic trend (GEE: χ^2^_1_ = 42.7, N = 10, p<0.001; Fig. 2D). The mean aggregation index peaked 54 h after hatching at 2.6, corresponding to ∼5 individuals in one quadrat. Ontario and lab-population larvae showed similar aggregation behavior, with no significant main effect of population (GEE: χ^2^_1_ = 1.3, N = 19, p = 0.258; Fig. 2E). Dishes of the Ontario and lab populations reached average maximum indices of 2.3±0.1 and 2.7±0.4, respectively, corresponding to ∼4 larvae in one quadrat. We observed a significant overall effect of time and a significant interaction between time and population (χ^2^_11_ = 562.0, p<0.001, and χ^2^_11_ = 34.7, p<0.001, respectively), indicating that the aggregation behavior of the two populations changed differently throughout development (see Fig. 2E). However, analyzing both populations independently revealed that both had a significant quadratic trend (*Canton S*: χ^2^_1_ = 11.0, N = 9, p = 0.001; and *Ontario*: χ^2^_1_ = 9.5, N = 10, p = 0.002), where the tendency to aggregate increases before declining prior to pupation. This suggests that the two populations exhibited similar general patterns of aggregation.

### Between Species Comparison of Larval Aggregation

We tested whether larvae of two closely related species, *D. melanogaster* and *D. simulans*, exhibit similar patterns of larval aggregation on standard food. There was a significant effect of species, with *D. melanogaster Canton S* larvae exhibiting greater aggregation than *D. simulans* during the latter stages of development, 50–80 h (GEE: χ^2^_1_ = 6.3, N = 20, p = 0.012; Fig. 2F). When analyzed separately, both species showed a significant quadratic trend (*Mel*: χ^2^ = 29.46, N = 10, p<0.001; and *Sim*: χ^2^ = 55.552, N = 10, p<0.001).

### Larval Aggregations and Improved Burrowing Ability

**Figure 3:**
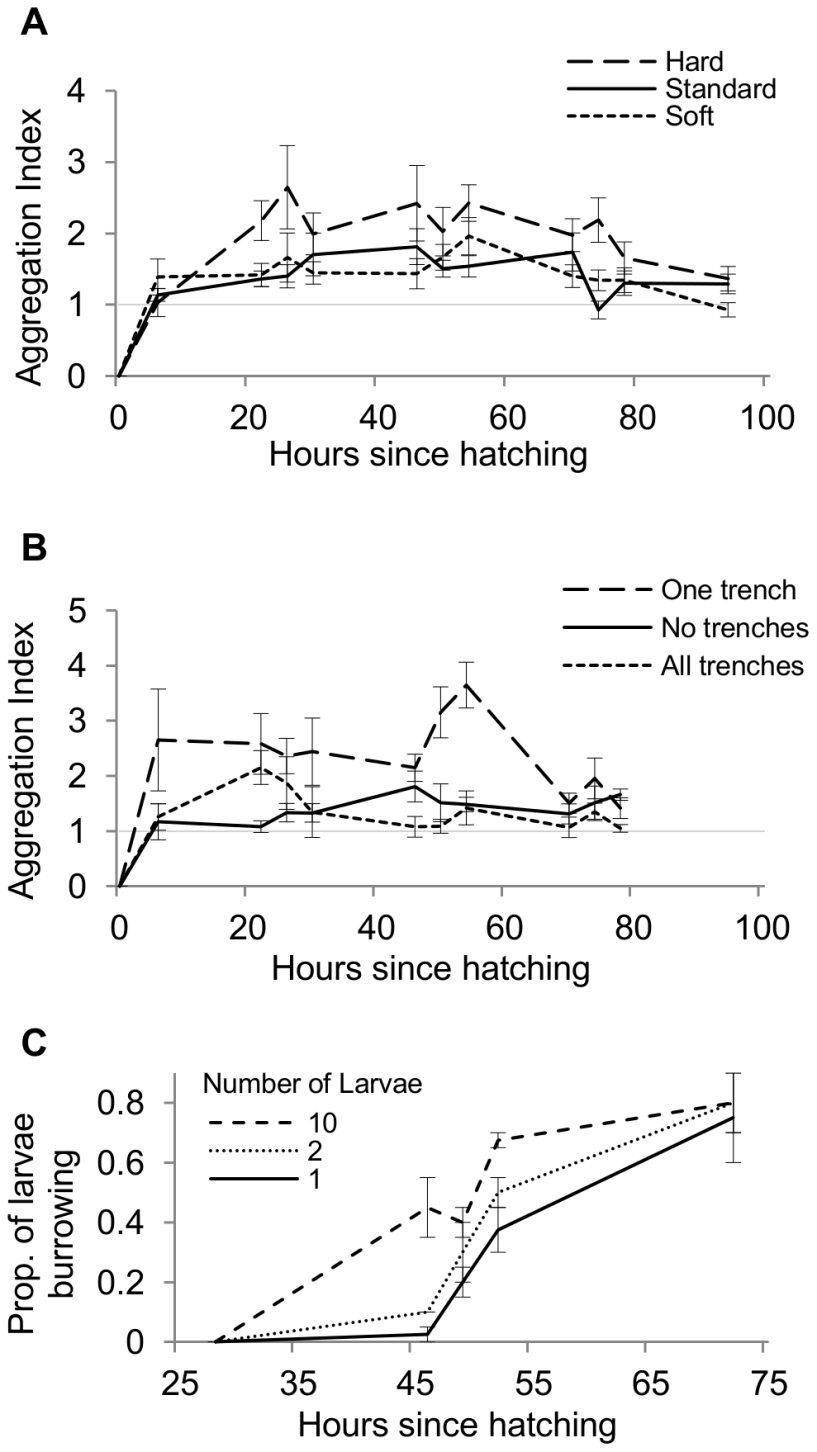
Aggregations and Burrowing. (**A**) Larvae exhibited greater aggregation behavior on harder substrates than on standard or soft substrates (N = 12 each), and (**B**) aggregated more at sites where the surface had been broken with an artificial “trench” (see methods, N = 7) than sites that had no trenches (N = 6) or a trench in all quadrats (N = 7). Note that even when food was uniformly soft or trenched at every quadrat, larvae still showed significant aggregation. Additionally, (**C**) when we manipulated the number of larvae per dish, larvae in larger groups started burrowing sooner than larvae in smaller groups (N = 6).

In the previous experiments (Fig. 2), larval aggregation peaked in late-second and early-third instar (approximately 40–80 hours after hatching). In these experiments, this roughly corresponded with the onset of larval digging and burrowing behavior, when larvae break the surface of the food and spend less time crawling. We tested whether this ability of larvae to burrow into the food substrate affected their tendency to aggregate. We predicted that, if larvae aggregate in order to improve burrowing, when the food is harder and therefore more difficult to penetrate, we would see increased aggregation. Conversely, we predicted that when the food is softer and easier to dig, we would see decreased aggregation. We modified the hardness of the food substrate by altering the concentration of agar in the recipe. In this and all subsequent experiments, we used *Canton S* strain larvae. Food hardness had a significant overall effect on aggregation (GEE, effect of food hardness: χ^2^_2_ = 17.8, N = 36, p<0.001; Fig. 3A), with larvae on harder food forming significantly greater aggregations than larvae reared on standard food (p<0.001) and softer food (p = 0.002). Larvae on hard food reached an average maximum aggregation index of 4.4±0.5 (N = 12), corresponding to ∼6 out of 9 larvae in one quadrat. Larvae in both standard and soft food treatments formed smaller but significant aggregations (compared to random distribution; Fig. 3A), which did not differ from one another (p = 0.801), reaching average maximum aggregation indices of 2.6±0.2 (N = 12) and 2.7±0.3 (N = 12), respectively, corresponding to ∼5 out of 9 larvae in one quadrat. There was a significant interaction between food hardness and time (χ^2^_22_ = 165.4, N = 36, p<0.001), but in all three food conditions: hard, standard and soft, there was a significant effect of time (all p<0.001), and a significant quadratic trend (all p<0.001; Fig. 3A), just as in previous experiments.

Next, we tested whether breaking the surface of the food *per se* affected aggregation behavior. We predicted that, if larvae aggregate in order to penetrate into the food (for instance, in order to hide from parasitoids), larvae given one quadrat with the surface already broken would show high levels of aggregation at this site, and conversely, larvae where every quadrat has the surface of the food already broken would show little aggregation. Breaking the surface of the food in one quadrat with a small “trench” (see methods) resulted in significantly greater aggregation than when all or none of the quadrats were trenched (p<0.001, both comparisons; GEE, effect of surface texture: χ^2^_2_ = 54.9, N = 20, p<0.001; Fig. 3B), with most larvae aggregating in the one trenched quadrat. Aggregation in the all- and none-trenched dishes did not differ (p = 0.541). Aggregation indices in the one-trench condition reached an average maximum of 5.2±0.4 (N = 7), corresponding to ∼6–7 larvae in one quadrat.

Finally, in a follow up experiment where we varied the number of larvae per dish, we found that group size significantly affected larval burrowing latency (GEE: χ^2^_2_ = 73.1, N = 6, p<0.001), with larvae in groups of ten burrowing sooner than either pairs or singletons (p = 0.001 and p<0.001, respectively; Fig. 3C). Pairs of larvae began burrowing sooner than singletons, but this difference only approached significance (p = 0.090).

### Detailed Behavioral Observations

**Figure 4:**
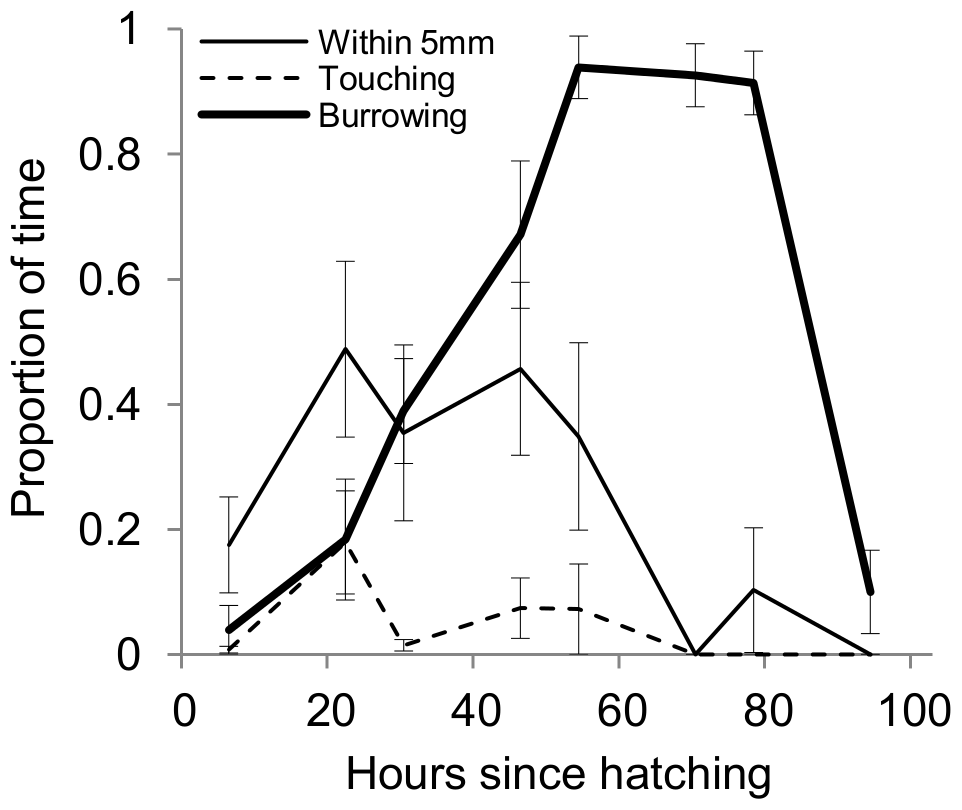
Detailed Behavioral Observations. We monitored two foraging larvae for ten minutes twice per day from hatching until pupation (N = 10 pairs). Larval social interactions, as measured by time observed within 5 mm of each other (thin solid line), and the time spent physically touching one another (dashed line) increased, then declined at 70 h post hatching. The proportion of time that larvae spent burrowing into the food (thick solid line) increased steadily to almost 1, before declining prior to pupation.

In order to better understand larval social interactions, we conducted detailed behavioral observations of pairs of larvae from egg to pupation. Two measures of social interaction, the proportion of time the two larvae spent within 5 mm of each other and the proportion of time larvae were in physical contact with one another, were greater in the first two days before falling at 70 h post-hatching (Fig. 4). This pattern is consistent with the aggregation indices of previous experiments. Additionally, larvae touched each other approximately once per 10 minute observation session for the first two days after hatching, but never touched after 70 h post-hatching (Fig. 4). Larvae typically found each other and then remained mobile before burrowing, crawling within 5 mm of each other in 2.2±0.4 quadrats across the experiment (N = 10). Burrowing behavior increased steadily, with larvae spending almost all of their time digging from 50 h after hatching until burrowing declined dramatically with the onset of wandering (Fig. 4).

## Discussion

We have developed a novel protocol for quantifying the dynamics of social behavior in a widely-used model organism well-suited for future investigations on the mechanisms, ecology and evolution of social behavior. Social behavior has been studied in a variety of insect larvae [26], [27], most notably, moth caterpillars including the tent caterpillar, *Malacosoma americanum* [28]–[30]. Nevertheless, our new protocols for quantifying the dynamics of social behavior in fruit fly larvae open up unique opportunities owing to the ample knowledge base and research tools available for *Drosophila* in general and *D. melanogaster* in particular [31]–[35]. Examples for such new opportunities include research on the ecology, evolutionary biology and neurogenetics of social information use [16], [36], the role of social behavior in defence against parasitoids [37], [38] and interactions with bacteria and fungi [21], [39], [40].

We have shown that larvae form modest foraging aggregations of four or five out of nine individuals, with social interactions peaking in the late-second-instar stage, regardless of initial distribution (Fig. 2B & C). These aggregations are not simply due to larvae preferring one site or quadrat of the dish, and form in different quadrats over time, suggesting that the larvae are not merely aggregating at the best site in their local environment, or a site that has been improved by others. Even a pair of larvae placed in a relatively large (9 cm^2^) dish will crawl alongside each other through multiple quadrats, often physically touching (Fig. 4), which suggests cooperative foraging. To our knowledge, this is the first documentation of such social behavior among fruit fly larvae.

Additionally, we have shown several lines of evidence indicating that larval social behavior allows for improved efficiency of burrowing, which can enhance fitness (see below). Larvae aggregate more on harder substrates (Fig. 3A), and more in sites where the surface has already been broken and thus is easier to dig (Fig. 3B). Pairs of larvae spend 40–50% of their time within 5 mm of each other until 70 h after hatching, and this corresponds to a steady increase in burrowing behavior (Fig. 4). Finally, groups of larvae initiate burrowing more quickly than larvae either in pairs or alone (Fig. 3C). Taken together, these results suggest that larvae may benefit from forming social foraging groups with an improved ability to dig into the substrate. Interestingly, other reports of cooperative digging and burrowing have observed the behavior during the wandering stage prior to pupation [13], [14], whereas our larvae typically exhibited a reduction in aggregation and burrowing behavior at this time.

Although forming aggregations will likely increase the level of foraging competition among larvae [16], this will be at least partially offset by improved burrowing, which may be important to the larvae for several non-mutually-exclusive reasons. Probably the greatest benefit that burrowing affords is an ability to hide from parasitoid wasps. Larval mortality from parasitoids can be enormous (up to 90% in some instances, [41]), and burrowing allows larvae off the surface of the food where they are most vulnerable to some species of parasitoids [42]. Our results are consistent with Rohlfs and Hoffmeister [43], who noted that greater densities of larvae were associated with an increase in the frequency of larval burrowing and a reduction in parasitism. Second, burrowing may allow larvae to better maintain homeostasis [20]. In particular, the temperature and humidity inside a fruit are much less variable than those at the surface. Third, larval burrowing may serve to break down and soften food, making it easier to ingest. Finally, larval burrowing may function to churn the food substrate, which can fight off competitive mould growth [44], [45], and can facilitate the growth of beneficial yeast species [21].

An improved ability to burrow, however, is not the only reason for larval aggregations. We observed aggregations even when foods were uniformly very soft and easy to penetrate (Fig. 3A), or uniformly pre-dug (Fig. 3B), indicating that larvae form small aggregations even when they can dig alone. This is consistent with our previous work in which we have suggested that larvae may benefit from copying the site choices of others, using the presence of others as social information to find higher quality sites [16]. Although in these experiments all quadrats were of equal quality, larvae may have modest, innate attraction to others even when the site currently occupied is of sufficient quality.

Finally, our data suggest that social behavior varies between species, as *D. melanogaster* exhibited a greater degree of aggregation than *D. simulans.* These two species co-occur in nature [41], [46], yet have been shown to exploit slightly different ecological niches [47]. We have proposed that one of the primary benefits of such burrowing is that it allows larvae to hide from parasitoid wasps. Interestingly, these two species differ in their defenses against parasitoid wasps, with *D. simulans* possessing greater physiological immune responses to parasites whereas *D. melanogaster* utilizes more avoidant behavioral defenses [38]. It could be that the greater larval sociality observed among *D. melanogaster* larvae serves to increase burrowing ability as a means to reduce parasitism. *D. simulans*, in contrast, may rely on their stronger immune system responses, and so avoid the competition costs associated with social foraging [16]. Interestingly, parasitoid wasps of different species may employ different searching strategies such as *vibrotaxis* (sensing the vibrations of larvae) and *ovipositor searching* (probing the substrate frequently in search of larvae) [48]. As Carton & Sokolowski [48] point out, digging is an effective strategy against wasps utilizing vibrations because a burrowed larva moves much less. On the other hand, burrowing makes it easier for *ovipositor searching* wasps to locate larvae, and in this case a better strategy may be increased mobility. It would be interesting to see whether larvae from populations or species that are exposed to wasps exhibiting different search strategies differ in their degree of sociality and burrowing.

In general, larval behaviors have been less well-studied than those of adults, yet for many researchers the larvae may prove a simpler and more tractable model system. For the study of social behavior in particular, the quantification of sociality among adults typically requires complex apparati due to the adults’ greater mobility and flight capabilities [49], [50], sometimes also including advanced computer tracking programs [51]–[53]. Our protocol for the quantification of larval sociality is simple and can further research into the evolution, ecology, and mechanisms of social behavior.

## Materials and Methods

### Fly Populations and Egg Collection

We used two populations of flies that have been maintained in our laboratory for several hundred generations (*D. melanogaster Canton S* and *D. simulans*), and one population of wild type *D. melanogaster* founded from a few hundred naturally occurring flies caught from several locations around southern Ontario using plastic bottle traps with slices of banana seeded with a sprinkle of active dry baker’s yeast. We tested this recently-caught population about 8 months after collection. For all populations we maintained several hundred flies in large cages on abundant standard food, one liter of which contained: 60 g dextrose, 30 g sucrose, 32 g yeast, 75 g cornmeal, 20 g agar and 2 g methyl paraben dissolved in 20 ml ethanol, in an environmental chamber at 25°C, 60% relative humidity, and on a 12∶12 light/dark cycle with lights on at 1 am. This light cycle placed peak egg laying midday so that we could collect eggs within a short window of time by providing flies with an 85 mm petri dish containing 10 ml standard food. Since females may hold developing embryos while searching for a suitable egg-laying substrate, prior to experimental egg collection, we provided females with a fresh food dish with a sprinkle of live yeast for 1 h, which we discarded. We then collected eggs for experimental larvae within 1 h on dishes without live yeast. We immediately transferred these eggs one at a time to experimental dishes with a soft paintbrush, taking care not to damage the eggs or the surface of the food. For aggregation assays, we placed one egg with its respiratory filaments facing up in the center of each quadrat, or, in the case of the perfectly aggregated initial distribution, all nine eggs in one quadrat. For detailed behavioral observations we transferred one egg each into two randomly selected side quadrats. All experimental dishes were stored in incubation chambers maintained at 25°C, high humidity, and total darkness throughout the experiments. We conducted all manipulations under red light, which larvae cannot see [54]. On the following day, we replaced any unhatched eggs (typically less than 20% of eggs per experiment, which may have been damaged or slower to develop) with age-matched larvae in order to keep the number of larvae per dish constant. In a preliminary experiment, we observed no differences in social behavior between larvae kept under red light and on a 12∶12 light/dark cycle (GEE: Wald χ^2^_1_ = 1.682, N = 20, p = 0.195).

### Aggregation Assay

We utilized a novel behavioral assay which allowed us to quantify larval social behavior over time. We filled a 3 cm×3 cm×2 cm (width×length×height) Plexiglas dish with 9 ml of standard food, 1 cm thick with a smooth, uniform surface. Each dish was covered with loose-fitting lid, which allowed some airflow. By marking the lid, we divided the dish into nine equally-sized 1 cm×1 cm quadrats (eg. Fig. 1). Beginning at 5 pm on the day following egg laying (6 h following hatching), we counted the number of larvae in each quadrat 3 times per day (9 am, 1 pm, and 5 pm) until pupation. Larvae moved freely throughout the experiment, and we took care not to disturb them during observations. In cases where larvae were crossing between quadrats at the time of observation, we recorded the location of their mouth. In the very rare case where this was still ambiguous, we watched the larva for a few seconds until it chose one quadrat. Larvae that had burrowed into the food could be identified by their posterior spiracles which remain exposed.

For each dish and time, we calculated an Aggregation Index (AI), defined as the variance-to-mean ratio [24]. We compared larval aggregation to the null model of random motion, defined by a Poisson distribution where the mean equals variance, AI = (variance/mean) = (1/1) = 1. Indices significantly greater than 1 indicate aggregated or “clumpy” distributions, and indices significantly lower than 1 indicate uniform distributions. Note that with this protocol, AI ranged from 0, a perfectly uniform distribution (one larva per quadrat), to 9, a perfectly aggregated distribution (all larvae in one quadrat). Due to violations of normality associated with our Aggregation Index, we tested the dynamics over time with Generalized Estimating Equations (GEE) with a gamma distribution and log link function [55]. In all experiments, *time* was included as a within-subject factor. Wald χ^2^ values are reported for these analyses. In experiments where larvae were initially placed in a uniform distribution, the expected climb in AI from 0 to 1 due to random motion could have resulted in spurious trends, so we modified indices from this time point to a value of 1 for analyses, which represented the null hypothesis of random motion. In cases where we observed a significant effect of time, we conducted trend analyses to see how the aggregation scores changed over time. Additionally, to get an idea of the typical peak of aggregation, we calculated the average maximum aggregation score from each dish whenever it occurred, reported as the ‘average maximum’ index in text.

### Quantifying the Dynamics of Larval Social Behavior

We examined how the two extreme initial starting distributions affected the dynamics of larval aggregation. The starting distributions were either uniform (one egg in each quadrat), or aggregated (all eggs in one quadrat). We first analyzed the initial distributions separately, and seeing that the Aggregation Index of the two rapidly converged within the first 22 h after hatching (Fig. 2B & 2C), we compared the pattern of aggregation from this convergence onward. We conducted additional analyses to assess two alternative hypotheses: (a) that larvae are merely attracted to one particular site in the dish (eg. the corners), or one particular quadrat in the dish (eg. center) due to external environmental factors, and (b) that larvae are not directly attracted to one another *per se* but form aggregations as a result of one larva improving a quadrat, which others then find attractive. First, we compared the sites of greatest aggregation, defined as the site where we observed the highest number of larvae. In the event that a dish had multiple sites with the same maximum of aggregation, we chose the quadrat with the greatest total number of larvae throughout the experiment. We compared the frequency of types of quadrats (corners, sides, or the middle) to the distribution expected by random chance (4∶4∶1, respectively) with a chi-square goodness of fit test. We compared the frequency of particular quadrats of greatest aggregation (numbered 1–9) with a “meta”-aggregation analysis where we compared the different locations of greatest aggregation to our null hypothesis of random distribution, similarly defined as an index of dispersion equal to 1. Larval dishes from both initial distributions had similar results from our “meta”-aggregation analysis, so we combined their results. Finally, to test whether larvae are not merely attracted to one higher quality site in the dish, we noted the total number of quadrats per dish where larvae formed aggregations and counted the number of times an aggregation shifted quadrats throughout the experiment. For this analysis, we defined an aggregation as four or more larvae per quadrat because aggregations of five or more larvae were not sufficiently common for statistical analyses.

We conducted our comparison of larval aggregation between two closely related species, *D. melanogaster* and *D. simulans*, by monitoring dishes containing eggs from the two simultaneously. In this and all other experiments involving multiple treatments, we ran all treatments simultaneously and had observers blind to treatment identities recording the data.

### Dynamics of Larval Aggregation on Fruit and in Wild Flies

We monitored larval aggregation on natural fruit. We cut 3×3×1 cm slices of clean, ripe honeydew melon (*Cucumis melo*) and dipped them into 0.3% active dry baker’s yeast (*Saccharomyces cerevisiae*) solution (0.3 g per 100 mL) to simulate natural inoculation but keep all quadrats of similar quality. We placed these slices snugly into the test dishes, added eggs, and recorded larval aggregation as before.

### Larval Aggregation and Burrowing

We simultaneously monitored the aggregation of larvae on dishes where we altered the toughness of the food by changing the concentration of agar in our standard recipe. We tested (a) our standard food recipe, (b) food in which we doubled the agar, making the food harder and more difficult to penetrate, and (c) food in which we halved the agar, making it much easier for larvae to dig into (2%, 4%, and 1% agar weight/volume, respectively). The surface texture of the food was similarly smooth in all treatments. We placed one egg per quadrat and monitored dishes as before.

Having found an effect of the food hardness, we compared larval aggregation in dishes with different surface textures. We either (a) left the surface of the food smooth, as in previous experiments, (b) dug away the surface of the food, creating a shallow 0.5 cm×0.7 cm×0.2 cm (width×length×depth) “trench” in the center of one randomly selected side quadrat, or (c) dug trenches in all quadrats. Breaking the surface of the food with trenches increased the rate of larval development, probably due to the softer food beneath, which is easier to ingest, and so pupation in these treatments began several hours earlier than in previous experiments (Fig. 3B).

As a follow up experiment, we directly tested whether groups of larvae are quicker to initiate burrowing into the food substrate. We monitored the proportion of larvae burrowing into the food, defined as having mouthparts below the surface of the food and no crawling on the surface. This number of burrowing larvae was confirmed by two raters (Pearson correlation, r = 0.92, p<0.001), one of whom was blind to the purpose of the experiment, and their data combined. Larvae were sorted *a priori* into groups of 10, either placed individually each on their own dish, in pairs, or all on one dish. We analyzed these proportions with Generalized Estimating Equations with normal distributions and identity link functions.

### Detailed Behavioral Observations

We sought to monitor the larvae more closely to better understand their social interactions. For ease of observation, we placed only two eggs per dish, one each into two randomly chosen side quadrats. Starting six hours after hatching, we observed each dish closely for ten minutes twice per day (10 am & 5 pm) until pupation. We recorded the duration of time that larvae were within 5 mm (approximately two body lengths) of one another, which we chose to approximate social interaction, and whether larvae were moving along the surface of the food or digging. Additionally, we recorded the frequency and duration of time that larvae physically contacted one another. Durations of separate events were summed and converted to the total proportion of each ten minute session per dish engaged in the behavior.
